# Deformation of Bioinspired MXene-Based Polymer Composites with Brick and Mortar Structures: A Computational Analysis

**DOI:** 10.3390/ma13225189

**Published:** 2020-11-17

**Authors:** Shreyas Srivatsa, Paweł Paćko, Leon Mishnaevsky, Tadeusz Uhl, Krzysztof Grabowski

**Affiliations:** 1 Academic Center for Materials and Nanotechnology, AGH University of Science and Technology, 30-059 Krakow, Poland; kgrabow@agh.edu.pl; 2 Department of Robotics and Mechatronics, AGH University of Science and Technology, 30-059 Krakow, Poland; pawel.packo@agh.edu.pl; 3 Department of Wind Energy, Technical University of Denmark, 4000 Roskilde, Denmark; lemi@dtu.dk

**Keywords:** MXenes, biomimicry, micromechanical models, finite element method, brick-and-mortar structures, computational analysis, effective interface model

## Abstract

In this work, the deformation behavior of MXene-based polymer composites with bioinspired brick and mortar structures is analyzed. MXene/Polymer nanocomposites are modeled at microscale for bioinspired configurations of nacre-mimetic brick-and-mortar assembly structure. MXenes (brick) with polymer matrix (mortar) are modeled using classical analytical methods and numerical methods based on finite elements (FE). The analytical methods provide less accurate estimation of elastic properties compared to the numerical one. MXene nanocomposite models analyzed with the FE method provide estimates of elastic constants in the same order of magnitude as literature-reported experimental results. Bioinspired design of MXene nanocomposites results in an effective increase of Young’s modulus of the nanocomposite by 25.1% and strength (maximum stress capacity within elastic limits) enhanced by 42.3%. The brick and mortar structure of the nanocomposites leads to an interlocking mechanism between MXene fillers in the polymer matrix, resulting in effective load transfer, good strength, and damage resistance. This is demonstrated in this paper by numerical analysis of MXene nanocomposites subjected to quasi-static loads.

## 1. Introduction

The discovery of nanomaterials in the last few decades has led to numerous applications of these nanomaterials in the fields of battery technology [1], sensors [2,3], wireless communication [4], and shock absorption [5]. Various nanomaterials like carbon nanotubes (CNTs), graphene, molybdenum di-sulfide (MoS_2_), and boron nitride (BN) were used as fillers with polymer matrices to form nanocomposites with new desired functionalities. Graphene was the first two-dimensional (2D) nanomaterial discovered in 2004 [6]. Along with graphene, other 2D materials (MoS_2_ and BN) were also used for various nanocomposites [7]. In 2011, a new 2D material was reported, namely, MXenes (Ti_3_C_2_T_x_, where surface termination T_x_ can be –O, –OH or –F) [8]. Almost immediately, MXenes attracted a great deal of interest in various fields of applications due to their unique physical properties such as good conductivity [8], film-forming ability and good elasticity [9]. Moreover, it has been reported that MXenes are environmental friendly materials (low toxicity [10] and biodegradable [11]), thus showing their potential in biosensing applications.

MXenes are inorganic compounds of metal carbides or nitrides. The surface termination and highly electro-positive edges of MXene materials result in hydrophilic behavior [12]. The presence of metal atoms in MXene results in good conductivity [8]. The hydrophilic behavior exhibited by MXenes has an advantage over other nanomaterials such as unfunctionalized CNTs, which tend to form agglomerates resulting in unpredictable behavior (non-uniform distribution of CNTs within the polymer composite leads to uncertain elastic and electrical properties resulting in unpredictable stress-strain or strain-resistance behavior) [8,13]. Moreover, hydrophilicity allows MXenes to be uniformly dispersed within a polymer matrix, therefore enabling a highly repeatable fabrication procedure. On the other hand, mechanical properties of monolayer MXenes are reported to be better than commercially available reduced-graphene oxides (r-GO), which are used extensively for applications with graphene [9]. These advantages of MXenes over other conventional nanomaterials provide unique and promising opportunities to progress in the current state of the art in nanocomposites.

Similar to other nanocomposites, MXene monolayers (delaminated form or flakes) are often used as fillers with polymer matrix materials resulting in MXene Nanocomposites (MXNC) [13,14]. Though pure MXene films have good mechanical properties and conductivity [13,15], these are not chemically stable for a long duration due to oxidation [16]. Unfortunately, this affects the conductivity and mechanical properties of MXenes. The same literature work also indicates the use of polymer material as a matrix for MXene nanomaterials to reduce the oxidation rate. Varying the MXene-to-polymer weight fraction and fabrication processes [13,14,17] results in a wide range of electrical and mechanical properties, creating space for tailoring an MXNC for specific application. This complex design process requires numerous and costly experimental procedures to find the proper combination of process and material parameters for obtaining the desired functionality of composites. The recently demonstrated brick-and-mortar (or layer-by-layer) assembly process of MXenes with controlled polymer intercalation [18] provides a solution for the controllable tailor-made fabrication process. Thus, this specific structure of MXene forms the main scope of this work, where analytical and numerical methods will be used in order to model such structures.

Pre-design of MXNC using analytical and numerical models plays a major role in overcoming the challenge posed by extensive physical testing of nanocomposites with different nanocomposite constituents. Models not only help in estimating the effective (overall) nanocomposite physical properties but also aid in predicting the behavior (response) of the nanocomposite to various loading types and scenarios (e.g., stress-strain response, strain-resistance response etc.). Developing models for such nanocomposites involves complexity in geometry and material properties from the nano to macro scale due to the size- and scale-effect phenomenon observed in composites, calling for multiscale modeling approaches. Several multiscale modeling strategies, as well as numerical techniques dedicated for nano-, micro-, meso- and macro-scale, and coupling procedures exist. Among the coupling procedures, the hierarchical approach [19,20,21,22] of scaling microscale models to macroscale models and the concurrent approach of modeling [23] without scaling are popular. Despite a wide variety of existing numerical tools for predicting the mechanical properties of materials in multiscale, their application in predicting the behavior of MXenes is very limited. Among the approaches developed so far, work on nanoscale modeling and estimating mechanical properties of MXenes with molecular dynamic techniques can be mentioned [24,25]. Recently, some work on microscale modeling of MXene/Polymer nanocomposite has been reported that uses the finite element method to analyze such micromechanical models [26,27]. Therefore, noting that there is a very limited number of works on modeling of MXene nanomaterials, we develop microscale mechanical models for bioinspired nacre-mimetic assembly MXNC.

In this paper, we consider the bioinspired brick-and-mortar structures of MXene/Polymer nanocomposites. Biomaterials in nature have inspired engineers for ages to develop composite materials for various applications [28]. Among these bioinspired materials and related pre-design, there have been efforts to mimic the bio-composites and develop man-made composites with enhanced strength, toughness, elasticity, and damage resistance capabilities [29,30]. The biocomposites considered for pre-design in this paper is nacre (known as mother of pearls). Nacre has more strength and toughness than its main constituent material of calcium carbonate. Investigations of nacre over the years have led to micro and meso scale designs biomimicking the brick and mortar structure [31]. The studies into tensile deformation of nacre at micro and macroscales [32] have led to nacre-mimetic pre-design of nanocomposites [33,34]. The brick-and-mortar assembly of the nacre provides topological and structural assembly advantages (interlocking mechanism) for the biocomposite to have greater strength and toughness than its main constituent [35]. We utilize these design benefits and develop pre-design configurations for MXene/Polymer nanocomposites. The paper provides a novel pre-design approach of micromechanical modeling and analysis study incorporating the bioinspired nacre-mimetic assembly of MXene/Polymer nanocomposites.

A literature review of the modeling of nanomaterial-based composites and particularly MXene nanocomposites emphasizes the need for a multiscale modeling approach to nanomaterial-based composites. The MXene nanocomposites developed over the last few years have indicated a stacking assembly of MXenes in a polymer matrix. Modeling methods that provide consistent elastic property estimation need to be explored. Pre-design and modeling of MXene nanocomposites for bioinspired nacre-mimetic assembly has not been explored in the literature yet, and so it is expected to provide better mechanical properties with effective load transfer between MXene fillers and polymer matrix through such work. Moreover, microscale model could potentially be included in further studies involving multi-scale approaches (such as nano and macro scales).

The structure of this paper is organized and discussed henceforth. First, the geometric and material properties of MXenes (titanium carbide, Ti_3_C_2_T_x_) are summarized based on a literature review outcome along with the experimental characterization tests carried out by the authors (provided in Section 3.1). Then, approaches for modeling a single MXene layer (flake) and MXene/polymer nanocomposite based on nacre-mimetic structure are discussed. MXNC films formed via brick-and-mortar (layer-by-layer) [33] assembly (same models can be applied to vacuum-assisted filtration [13,14,36] process formed MXNCs but the stacking and layer-by-layer formation is not controlled) are the focus of these model developments. The consistent stack formation of MXene with polymer material provides the basis for the deterministic model configurations considered later. Subsequently, analytical and numerical methods with potential applicability in terms of modeling MXNC—like effective interface model (EIM) [37], classical laminate plate theory (CLPT) [16,38], and finite element analysis (FEA)—are discussed and implemented. Finally, a comparison is made between the reported experimental results and results obtained from all the MXNC models employed in this paper. This leads to discussions on the use of the brick-and-mortar assembly during fabrication of MXNC and their influence on the effective nanocomposite behavior. The paper concludes with a discussion of the results.

For the purpose of modeling and property estimation, weight fractions of MXenes and polymer will be used throughout this paper. Weight fraction can be converted to volume fraction based on the density of the filler and matrix, and the volumes depend on the models developed in this paper. The units used in the paper are mm, N, t/mm^3^ unless otherwise stated.

## 2. Modeling and Methods

### 2.1. Mechanical Properties of MXenes and Polymers

#### 2.1.1. MXenes

Geometrical properties of MXene (Ti_3_C_2_T_x_) monolayers have been studied extensively. The results [9,11] of morphology and characterization of MXenes (Ti_3_C_2_T_x_), developed in the last few years obtained using a synthesis process of in-situ hydrogen fluoride (HF) formation with the minimally intensive layer delamination (MILD) method, indicate an average lateral dimension of MXene (Ti_3_C_2_T_x_) monolayers to be in the range of a few microns (1–12 µm, without sonification) and the thickness to be in the range of few nanometers (1–10 nm) [39]. Lateral dimensions of 2 microns and a thickness of 2 nm is used for all the MXene monolayer models in this paper.

The elastic properties of the MXene monolayer are determined through experiments like nanoindentation using Atomic Force Microscopy [9] and various computational processes in literature on Molecular Dynamics (MD) [16,24] and Density Functional Theory (DFT) [40,41] were used. The Young’s modulus of MXenes (Ti_3_C_2_T_x_) estimated using DFT, molecular dynamics, and experimental results are 312.5 GPa (with Poisson’s ratio of 0.2265), 502 GPa and 330 ± 30 GPa, respectively. As the prediction of DFT studies are close to the experimental results, we consider the material properties from the DFT studies in this paper (provided in Table 1).

#### 2.1.2. Polymers

Two polymers are considered in the process of analysis in this paper, namely, epoxy-resin and polyvinyl alcohol (PVA). The material properties of PVA are: Young’s modulus of 1 GPa, Poisson’s ratio of 0.42, density of 1.19 × 10^−9^ tmm^−3^ and allowable maximum stress of 30 MPa. The material properties of epoxy-resin are: Young’s modulus of 3.0741 GPa, Poisson’s ratio of 0.29, density of 1.1 × 10^−9^ tmm^−3^ and allowable maximum stress of 49.9 MPa [13,33,39,42,43].

### 2.2. Analytical Methods

**Figure 1:**
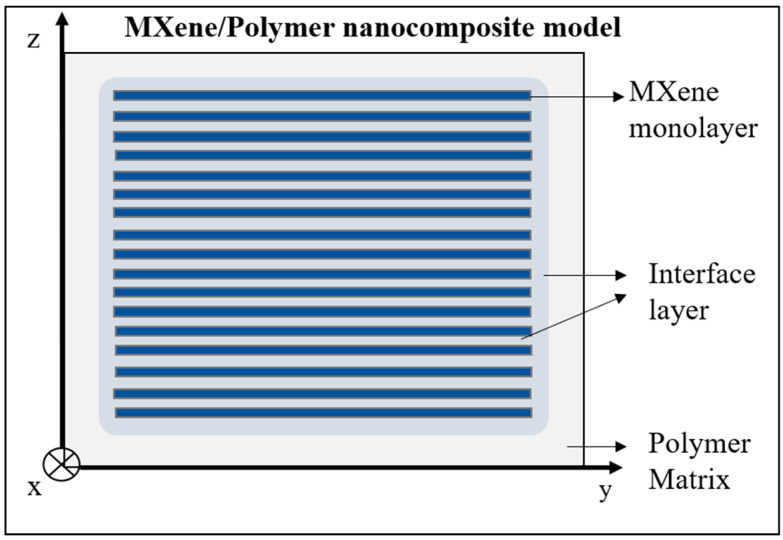
MXene/Polymer nanocomposite model representation at micro scale without MXene overlaps and coordinate axes of X, Y, and Z, which are orthogonal to each other.

Once the properties of single-layer MXene model are defined, models for MXNC are built by distributing several flakes within the polymer. The topology is based on deterministic configurations derived from MXNC samples fabricated by brick-and-mortar (layer-by-layer) assembly (a similar method can be used for vacuum-assisted filtration fabricated samples as well). The polymer is modeled as a representative volume cube within which the MXene monolayers are orderly distributed by defining the weight fractions of each constituent in the composite used in the analytical methods. The analytical methods of EIM and CLPT—used in this study—consider the interface between a MXene monolayer and polymer. A generic MXNC model with a set of MXene flakes with polymeric matrix is shown in Figure 1. The model assumes the interface layer to have the same shape as the filler.

There are two interfaces defined in this paper—namely, the filler-matrix-filler interface (in case of a stack formed with MXenes and polymer) and the filler-matrix (between MXene monolayer and polymer) interface. Both these interfaces are assumed to have the same elastic properties for the EIM and CLPT method. The analytical models help in studying the effective MXNC elastic properties and comparing them with the reported experimental results.

Effective interface model: The Effective Interface Model is a modified approach of the continuum mechanics-based Mori-Tanaka model [37]. The latter is based on analytical considerations of Eshelby’s inclusion principle [44,45]. The model considers the filler, matrix, and interface material of the MXNC. In determining the mechanical response of the MXNC, MXene monolayers are assumed to be distributed in an infinite space of polymer matrix material. With this assumption, the MXene flake is considered as the inclusion in the current model [46]. Based on the dimensions of MXenes considered in Section 2.1.1, the MXene flake is approximated according to Eshelby’s inclusion as a penny shaped inclusion (L = B> > h) for the purpose of forming Eshelby’s tensor, which is essentially a tensor based on geometric properties of the inclusion or filler (in this case, the MXene flake). Here the filler-matrix-filler interface and filler-matrix interface are assumed to have the same properties as previously discussed. The drawback of the method is that a single filler material with interface is assumed in an infinite matrix space around it, thus, the stacking and MXene assembly effect due to the fabrication process cannot be captured from this model. The equations for EIM formulation are provided in Appendix A. 

Classical laminate plate theory: Although a single MXene layer behaves like a membrane, the resulting bioinspired nacre-mimetic MXNCs display mechanical properties that may be effectively modeled via continuum-based approaches for plates. This is valid for a stacked sequence of MXene flakes embedded in a polymeric matrix, making such a composite setup very similar to a multilayered composite (shown in Figure 1). The MXNC is modeled using the CLPT to estimate the effective elastic properties of the MXNC and incorporate the interface layers (and provide estimations of their elastic properties). Here, the Kirchhoff’s Plate theory—assuming the normal material line being infinitely rigid along its own length, normal material line of the plate remaining a straight line after deformation, and normal material line being normal to the deformed plane of the plate—is considered for modeling MXNCs [38]. The method considers MXene flakes as thin plates (with small displacement or rotations and small strains) with polymer material between them. The assumptions for displacements and strains leading to the equations are provided in Appendix B. The in-plane stiffness matrix of the formulation provided in Appendix B is considered for this paper as we only consider tensile loading of the MXNC model.

### 2.3. Numerical Methods

**Figure 2:**
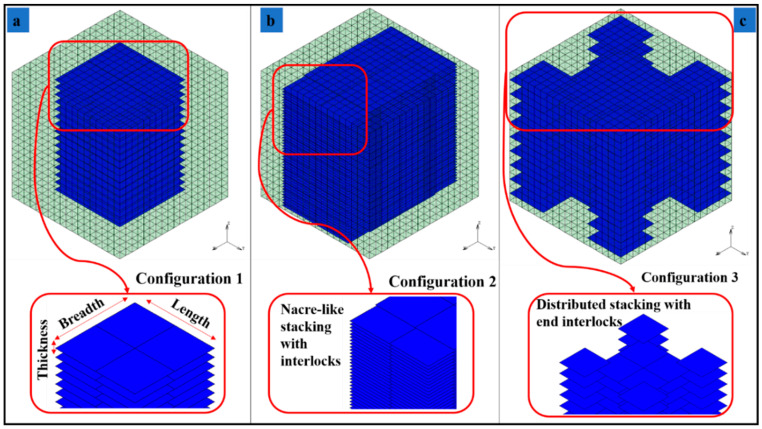
RVE configurations of MXene/Polymer nanocomposite. (**a**) Configuration 1, (**b**) Configuration 2, (**c**) Configuration 3.

The limitations of analytical modeling techniques related to shapes, thicknesses, distribution, interactions between the inclusions and other aspects lead to the application and developments of numerical methods for predicting mechanical responses of MXenes and bioinspired MXNCs. A common choice is the widely used and versatile finite element method. Among many tools and techniques available in finite elements, the so-called multi-point constraints (MPCs) can be effectively used for modeling MXenes with polymer material. In the proposed numerical model, MXene flakes and the polymer matrix are meshed independently, while the two sets of meshes do not need to be congruent (i.e., no common nodes are required). Next, the nodes of the MXene flakes are tied to the nearest nodes of the polymer matrix via MPCs. This allows the field variables (displacements, temperatures, currents etc.) at nodes of MXene flakes to be linked with the field variable at the nodes of the polymer. For setting up the MPC equations, the interface is assumed to be perfectly bonded between the filler (MXene) and matrix (polymer). Figure 2a–c illustrates the configurations used for modeling MXNC. These topological distributions are based on the bioinspired nacre-mimetic brick-and-mortar assembly. Configuration 1 consists of a simple MXene/Polymer nanocomposite model, while configuration 2 and 3 utilize nacre-mimetic nanocomposite assembly. The MXene/Polymer nanocomposites, modeled as RVEs [47,48] with a cube configuration at the micro scale, can later be used for hierarchical material framework for multi-scale analysis. A representative volume cube of 3.3 µm as the side length is considered.

Element selection: A single MXene layer in the RVE is modeled using thick plate elements. The current models were implemented in MSC Marc software; therefore, the shape function details are provided in [49]. The material properties used for the model are given in Section 2.1. Unlike the CLPT analytical model, which has a thin plate assumption, the thick plate element in the numerical model uses a modified version of the Mindlin-Reissner plate model [50] whose original version is also sometimes referred to in literature as the First-Order Shear Deformation Theory (FSDT) [51]. The FSDT theory assumes the normal material line, initially normal to the mid-plane of the plate, remains straight and unstretched after deformation, but not necessarily normal to the mid-plane of the plate. This leads to the finite rotations of the cross-section of the plate to be considered. The present modification [50] of the thick plate element theory consists of the formulation of parabolic distribution of transverse shear strains and satisfies the zero transverse shear stress requirements on the plate surfaces. The set of assumptions about the strain of these thick plate elements is provided in Appendix C for further reference. The polymeric matrix is represented by eight-node three-dimensional brick elements with a trilinear interpolation. These elements have three global displacements as degrees of freedom at each node along with eight-point Gaussian integration. Details on the element’s shape function formulation are given in Appendix C. 

Boundary conditions: Numerical models of MXNCs are subjected to test conditions similar to experimental works in literature [13] in order to estimate their mechanical properties via a virtual tensile test [52]. Subjecting the microscale models in the form of RVE to a 1-D virtual tensile test, the scaling-up of microscale models to macroscale to analyze the elastic properties may be omitted and the results from the RVEs can be directly analyzed (following Saint-Venant’s principle). This helps in the study of micromechanical model response subjected to quasi-static uniaxial displacement boundary condition at one end and fixed boundary condition at the opposite end. 

Furthermore, for the estimation of elastic properties, particularly, the Young’s modulus of the MXNC from the RVEs, periodic boundary conditions along with the Hill-Mandel condition [48,53,54] are applied. This was implemented in MSC Marc through in-house Fortran subroutines and quantitative results are provided in subsequent sections.

## 3. Results and Discussions

### 3.1. Experimental Characterization of MXene Samples

**Figure 3:**
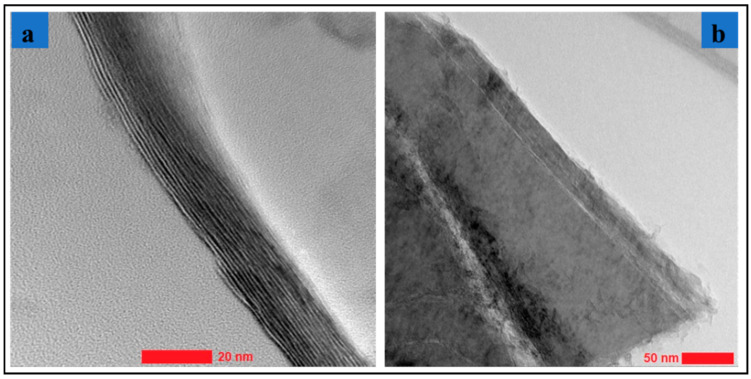
(**a**,**b**) HR-TEM images of stacks of pure MXene samples.

**Figure 4:**
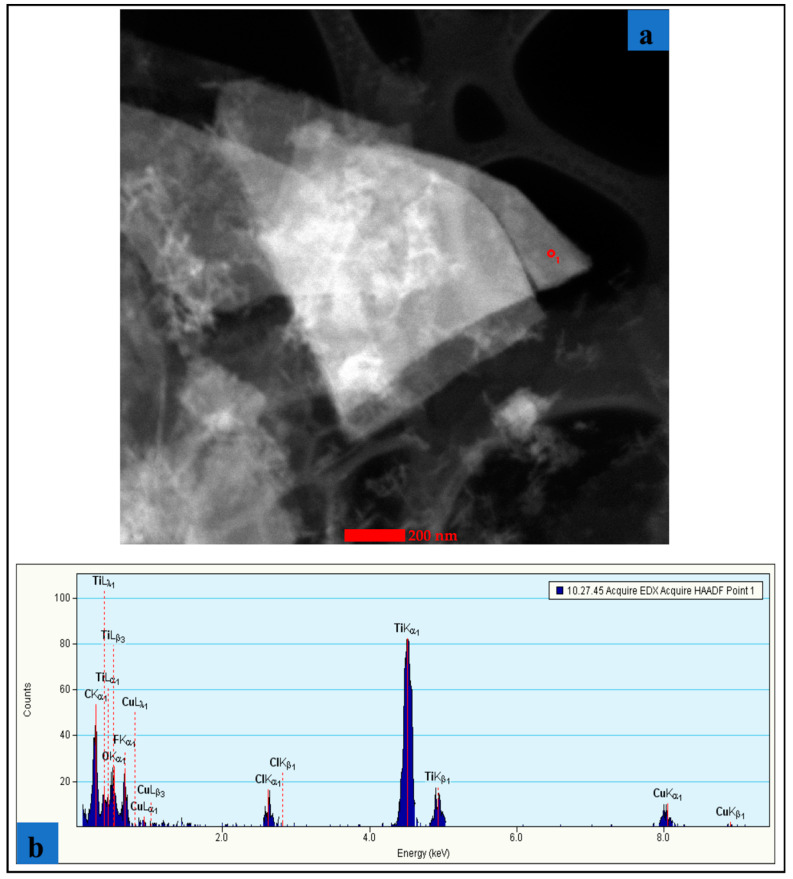
(**a**) TEM image of MXene flakes (**b**) EDX analysis of the elemental composition.

The MXene (Ti_3_C_2_T_x_; supplied by Materials Research Centre, Kiev, Ukraine) sample morphology and structure were investigated using transmission electron microscopy (TEM) (manufacturer details: Tecnai TF 20 X-TWIN, FEI Company (subsidiary of Thermo Fisher Scientific), Hillsboro, OR, USA) and scanning electron microscopy (SEM) (manufacturer details: Versa 3D scanning electronic microscope with field electron gun and ion, FEI Company (subsidiary of Thermo Fisher Scientific), Hillsboro, OR, USA). The lateral dimensions and single layer thicknesses of MXene (Ti_3_C_2_T_x_) were measured. Figure 3a,b show the stack of pure MXenes flakes observed using high resolution TEM (HR-TEM, FEI Company (subsidiary of Thermo Fisher Scientific), Hillsboro, OR, USA). The thicknesses of these MXene samples were approximately 1–2 nm, confirming other literature studies (a thickness of 1–10 nanometers and lateral dimensions in the order of 1–10 microns). Figure 4 provides energy dispersion X-ray (EDX, FEI Company (subsidiary of Thermo Fisher Scientific), Hillsboro, OR, USA) spectroscopic data of MXene samples confirming the presence of titanium carbide with surface termination (Ti_3_C_2_T_x_). Combining these results with the other geometrical features of MXenes (see e.g., [13]) the yielded data to be used for MXNC models is developed in Section 2.

### 3.2. Estimations of Effective Young’s Modulus of MXNC

The two analytical models are implemented for the various weight fractions of MXenes in polymer. The model parameters are shown in Table 2. Various deterministic configuration implemented for numerical models are marked (Table 2) for the same weight fractions. MXene/Epoxy-resin nanocomposite simulation results and the quantitative comparison of these results with reported experimental ones are shown in Table 3, while the results for the MXene/PVA nanocomposite are shown in Table 4.

Analytical models of EIM and CPLT are implemented based on their respective formulation in MATLAB code form. These models are tuned for the weight fractions of MXene in polymer. The total volume of these models is defined using a representative volume cube for the purpose of providing volume constrains (thereby, weight constraints) on the models developed. The number of MXene layers depend on the weight (volume) fraction of MXene and is given in Table 2. The interface volume fraction is defined as a function of the MXene weight fraction. Based on these two weights (volume) fraction values, the matrix weight (volume) fraction is calculated. These weight (volume) fractions are normalized for the purpose of generalization. Individual subroutine codes (in MATLAB code) are written for each analytical model and the results of stiffness matrix estimation is used to finally obtain the effective Young’s modulus values of the MXNC. The equations provided in Appendix A and Appendix B are implemented and the Young’s modulus is derived from the in-plane stiffness matrix for CLPT. The resulting estimations of the effective Young’s modulus from the EIM and CLPT for MXene/Epoxy-resin nanocomposite are an overestimation and underestimation, respectively, compared to the experimental results (literature-based) and numerical results (obtained in this paper). In the MXene/PVA nanocomposite case, both EIM and CLPT methods overestimate the effective Young’s modulus compared to other results. These estimations from the analytical methods clearly indicate oversimplification of the models developed using these methods. They are less effective unless they can be modified and the underlying assumptions can be improved to capture the physics (close to experimental conditions) of the material being modeled.

The numerical model consists of RVE built with 8000 (20 × 20 × 20) polymeric matrix brick elements created in finite element method. The number of MXene layers are chosen based on the weight fraction of MXenes defined by the configurations. The configurations given in Figure 2 and Table 2 are under consideration here. The MXene monolayer modeled as plate elements have their nodal field variable linked to the nearest matrix brick element nodes using MPCs discussed in Section 2.3. 

The results of numerical models are provided in Table 3b. For configuration 1, the increase of MXene weight fraction in epoxy-resin from 2.96 to 24.42% leads to an increase of the effective Young’s modulus values by 7.5321%. For the configuration 2, a decrease in effective Young’s modulus values by 1.66% is observed for the same change in weight fraction of MXene in epoxy-resin. Configuration 1 has a simple distribution of MXenes while configuration 2 has an edge overlap (interlock) nacre-mimetic brick-and-mortar assembly of MXenes. Configuration 3 models the nacre-mimetic brick-and-mortar assembly process with end interlock regions. Both configuration 2 and 3 can have controlled polymer interaction and this has been demonstrated by experiments in literature [33]. For configuration 3, the increase in weight fraction of MXene in epoxy-resin from 1.093 to 5.0709%, 5.0709 to 14.9518% and 14.9518 to 42.12% results in an increase in effective Young’s modulus of MXene/Epoxy-resin nanocomposites from 8.546 to 11.05705 and to 3.3659%, respectively. For an increase of MXene weight fraction from 1.093 to 14.9518% and 1.093 to 42.12%, the effective Young’s modulus of configuration 3 increases by 21.1105 and 25.1817%, respectively. The strength (maximum stress capacity within elastic limits) of the RVEs are given in Table 3c. In configurations 1, 2, and 3, with the increase in MXene weight fraction in epoxy-resin from 2.96 to 24.42%, 5.67 to 42.12%, and 1.093 to 42.12%, results in the increase in strength by 1.7209, 42.374, and 11.9081%, respectively.

**Figure 5:**
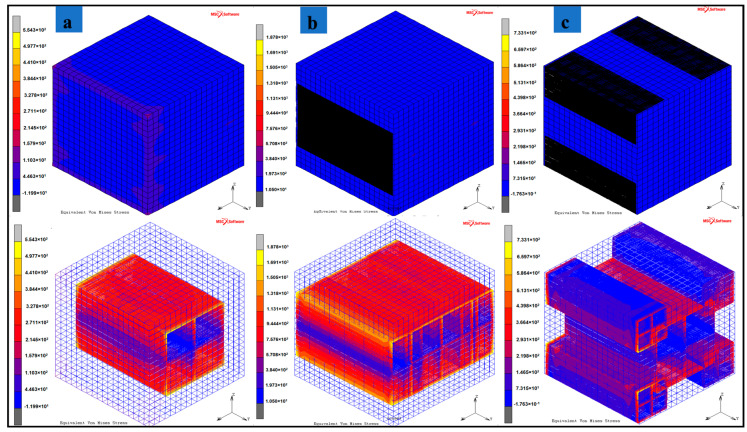
Stress field due to quasi-static tensile loading (X-direction) for various configurations (**a**) Configuration 1–24.42 wt.% of MXene in epoxy-resin, (**b**) Configuration 2–42.12 wt.% of MXene in epoxy, (**c**) Configuration 3–42.12 wt.% of MXene in epoxy.

**Figure 6:**
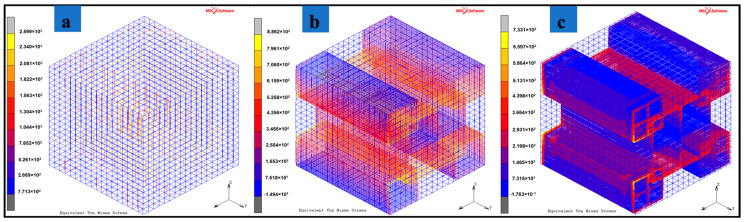
Quasi-static tensile loading (X-direction) applied to configuration 3 with different weight fractions of MXene in epoxy-resin. The resulting stress fields are shown in (**a**) 5.0709 wt.% of MXene in epoxy, (**b**) 14.9518 wt.% of MXene in epoxy, (**c**) 42.12 wt.% of MXene in epoxy.

Along with the increase in MXene weight fraction, the assembly pattern of MXenes in the epoxy-resin results in improving the load bearing capacity and effective transfer of load from the polymer material to the MXene filler material. The bioinspired nacre-mimetic brick-and-mortar configurations used in configurations 2 and 3 indicate these enhanced elastic behaviors of the MXene nanocomposite. Figure 5a–c indicates that these enhanced elastic behaviors in which the polymer experiences less stress, and the applied load is distributed among the MXenes according to the assembly pattern. Interestingly, configuration 2 has the MXenes stacked along the direction of the quasi-static load and not sparsely distributed as in configuration 3, and this results in greater strength for the RVE for configuration 2 (Figure 6 and Table 3b,c) provides illustrations of stress fields of the three configurations. The sparsely distributed configuration 3 also has an increase in strength as well as an increase in the effective Young’s modulus values. The interlocking mechanism between the MXene fillers due to the nacre-mimetic assembly allows for effective load transfer from polymer to MXenes and with increasing weight/volume fraction of MXenes, the strength and effective Young’s modulus increases (configuration 3 shown in Figure 6 & Table 3b,c). The nacre-mimetic brick-and-mortar assembly process offers the advantage of tailoring the MXene assembly with polymer intercalation and this ensures an effective load transfer, as seen from the results in Table 3c. With controlled assembly of the MXenes with the polymer, along with an increase in the load-bearing capacity, the damage propagation path might also be controlled. The crack propagation in such nanocomposites are along the fillers surfaces and their interfaces with polymer matrix [55].

### 3.3. Comparisons with Literature-Based Experimental Results

For MXene/Epoxy-resin nanocomposites shown in Table 3a,b, the EIM estimates the Young’s modulus values of the nanocomposite to be of an order of magnitude higher than the reported experimental results (for the weight fraction of MXene filler above 10%). The EIM results has overestimations compared to the reported experimental results [9]. The results from CLPT underestimates the effective nanocomposite Young’s modulus values by an order of magnitude lesser than reported experimental results (except for weight fraction close to 40 wt.%) as seen in Table 3a. The interface Young’s modulus employed in the EIM and CLPT methods is documented in Table 3a.

**Figure 7:**
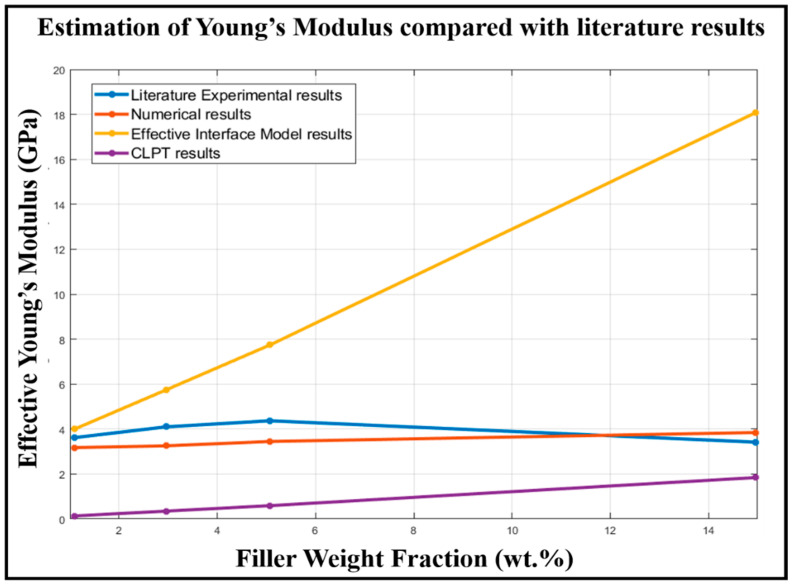
MXene/Epoxy-resin nanocomposite modeling results compare to reported experimental results.

The FEA results estimate the Young’s modulus values to be of the same order of magnitude as the reported experimental results. The FEA-based results provide a more consistent estimation close to the reported experimental results for the MXene/Epoxy-resin nanocomposite. The numerical results of configuration 3 shown in bold in Table 3b are compared with results presented in [14]. Error in estimation from numerical models in comparison with reported experimental results is in the range of 12.4–21.09%. The quality of estimation of Young’s modulus values in comparison with reported experimental results are shown in Figure 7. The numerical results show an increase in Young’s modulus values of MXNC with an increase in weight fraction of MXene in the composite. The reported experimental result indicates an increase in the Young’s modulus value, reaching a maximum value of 4.37 GPa and then a reduction in the Young’s modulus with respect to weight fraction of MXene in the composite. The numerical model results provide a more consistent prediction compared to the classical analytical models.

The same interface conditions presented in Table 3 are employed for the MXene/PVA nanocomposite results shown in Table 4. EIM, CLPT, and modified CLPT reached the volume constraint condition (restricting the fillers from being modeled outside the RVE volume) imposed on the RVE models in the MATLAB code and hence the weight fraction values were at 38.06% for EIM and CLPT and 38.09% for the modified CLPT method. The MXene/PVA nanocomposite numerical results are of the same order as those obtained experimentally in literature [8] but the reported experimental result value is more than twice that of the numerical one. 

The various analytical and numerical models developed and tested against the experimental results reported in this paper provide a wide range of possibilities for further model refinements. A designer based on the requirement can use the models during the process of pre-designing the MXNC. An estimation of nanocomposite properties from these models will allow the MXNC designer to select a model for composite properties simulation and compare it with the models developed in this paper. The advantages and drawbacks of these models concluded in this paper can support the design process.

### 3.4. Discussions on the Influence of the Interface between MXenes and Polymer

The assumption of a perfect bonding condition between MXene and polymers (in the numerical model) seems to be the cause of some variation in the estimation of the results in the case of epoxy-resin and PVA matrix-based nanocomposites. In both the cases, the surface termination of MXenes plays a crucial role in bonding with the polymer chain in the fabrication of MXNC and modeling it within the numerical model framework is an important step. The results also indicate that the numerically obtained Young’s modulus values of MXene/PVA nanocomposite are half of the experimental results obtained in literature. The increased strength of the composite compared with the pure PVA (an approximate Young’s modulus of 1 GPa) indicates a strong bond between the hydroxyl group of PVA polymer chain and the surface termination of MXene. The effect of the size of MXene monolayers on the composite and interface bonding also requires further investigation. The surface termination of MXene can lead to two types of bonding as per the chemical bonding study [56]. The surface termination of MXenes might feature either hydrogen or van-der-Waal type of chemical bonding with the polymers. MD simulations and experimental investigation of the effect of bonding can be adopted to the models presented in this paper. Several new MXenes [57] that are being discovered can be adopted to the models developed in this paper as well, particularly, the numerical models. Finally, the micromechanical models developed in this paper form a basis for a multi-scale study of MXene mechanical properties in the future. The models developed with numerical methods provide a consistent estimation of the Young’s modulus of the nanocomposites compared to the analytical methods.

## 4. Conclusions

The paper focuses on developing models for bioinspired nacre-mimetic brick-and-mortar assembly of MXene (Ti_3_C_2_T_x_) with polymer matrix. Nacre-mimetic composites have had a history of providing good strength, toughness, damage resistance, and effective load-bearing capacity. These advantages of nacre-mimetic assembly led to the development of the MXene/Polymer nanocomposite configurations discussed in this paper. An experimental characterization of pure MXene (Ti_3_C_2_T_x_) samples were made to study the morphology and dimensionality of MXene samples and validate the observations contained in the literature. The material properties of MXenes (Ti_3_C_2_T_x_) and polymers were determined from the literature. MXene micromechanical models are developed using a simple arrangement as shown in configuration 1 and with nacre-mimetic assembly (with interlocking mechanism at the edges of the MXenes) as shown in configuration 2 and 3. The models were analyzed by using two analytical methods, namely EIM and CLPT, with consideration of the interface regions between MXene and the polymer. Numerical models based on the FEM with the MPC method considered the perfect bonded condition between MXene and the polymer. The EIM overestimated the MXene/Epoxy-resin nanocomposite effective Young’s modulus value by an order of magnitude compared to reported experimental results (up to 40% weight fraction of MXene). The CLPT method estimated the MXene/Epoxy-resin nanocomposite effective Young’s modulus value to be of the same order of magnitude or an order lesser compared to the reported experimental results. The numerical results estimated the MXene/Epoxy-resin nanocomposite effective Young’s modulus values to be of the same order of magnitude as that of the reported experimental results and more consistent than the analytical methods. The estimation errors for the numerical models of the MXene/Epoxy-resin nanocomposite compared to the reported experimental results were less than 21%. The results indicate that better models for bonding conditions between MXene/Epoxy-resin and MXene/PVA may lead to close quantitative estimations to the reported experimental results. For configurations 1, 2, and 3, the increase in MXene weight fraction in epoxy-resin from 2.9 to 24.4%, 5.6 to 42.1% and 1 to 42.1% results in an increase in the effective Young’s modulus by 7.5, 1.6, and 25.1%, respectively.

The results provide an interesting insight that the nacre-mimetic brick-and-mortar assembly configuration of MXene with polymer intercalation can also play a role in controlling the elastic properties of the nanocomposite. This is also helpful in controlling the effective load transfer from the polymer material to the MXene filler material. With the three configurations, we observe that the nacre-mimetic configurations 2 and 3 with an interlocking mechanism assembly of MXenes with polymer have a different load bearing capacity and the strength depends on the assembly pattern. The controlled assembly of MXene with polymer intercalation can result in a better load-bearing capacity of the nanocomposite by effective transfer of the load to the MXene filler and the damage propagation path may also be controlled with further optimization of the brick-and-mortar assembly process.

## Figures and Tables

**Table 1:** MXene’s physical properties [40].

Mechanical Properties	Symbol	Values	Units
Young’s Modulus in planar direction	E	312.5	GPa
Poisson’s ratio in the planar direction	µ	0.2265	-
In-plane shear modulus	G	141	GPa
Density	ρ	~3.2 × 10^−9^	tmm^−3^
Maximum allowable Tensile stress	σ	(17.3 ± 1.6)	GPa

**Table 2:** Implementation of the three configuration models using the numerical method (Configurations and the corresponding weight fraction of filler is denoted by “X” mark.).

Volume Fraction of Filler vol.%	Weight Fraction of Filler wt.%	Number of MXene Layers	Configuration 1	Configuration 2	Configuration 3
0.37844	1.093	17			X
1	2.96	45	X		
1.8031	5.0709	81			X
2	5.67	91		X	
5.6988	14.9518	256			X
10	24.42	449	X		
20	42.12	899		X	X

**Table 3:** (**a**) MXene/Epoxy-resin nanocomposite analytical results; (**b**) MXene/Epoxy-resin nanocomposite numerical results compared with reported experimental results; (**c**) MXene/Epoxy-resin nanocomposite numerical results with maximum stress and strain for each configuration implemented.

(a)				
**MXene/Epoxy**	**Weight Fraction (wt.%)**	**E Experimental** [14] **(GPa)**	**EIM (GPa)**	**CLPT (GPa)**
Interface Layer E (GPa)			1 × 10^−3^	3.25 × 10^3^
	1.093	3.62	3.998	0.1378
	2.96	4.1	5.7463	0.3517
	5.0709	4.37	7.74	0.5941
	5.67	Not Available	8.3274	0.6654
	14.9518	3.42	18.069	1.8419
	24.42	Not Available	29.578	3.2179
	39.52	Not Available	52.295	5.8916
(b)				
**MXene/Epoxy**	**Weight Fraction (wt.%)**	**E Numerical (GPa)**	**E Experimental (GPa)** [14]	**Error in Estimation (%)**
Configuration 1	2.96	3.2554	4.1	20.60
Configuration 1	24.42	3.5006	Not Available	Not Available
Configuration 2	5.67	3.4028	Not Available	Not Available
Configuration 2	42.12	3.346	Not Available	Not Available
Configuration 3	**1.093**	**3.1769**	**3.62**	**12.24**
Configuration 3	**5.0709**	**3.4484**	**4.37**	**21.09**
Configuration 3	**14.9518**	**3.8474**	**3.42**	**12.50**
Configuration 3	42.12	3.9769	Not Available	Not Available
(c)				
**MXene/Epoxy**	**Weight Fraction (wt.%)**	**E Numerical (GPa)**	**Maximum Stress (MPa)**	**Maximum Strain**
Configuration 1	2.96	3.2554	63.92	0.0207
Configuration 1	24.42	3.5006	65.02	0.0204
Configuration 2	5.67	3.4028	57.37	0.0189
Configuration 1	42.12	3.346	81.89	0.0258
Configuration 3	1.093	3.1769	63.99	0.0202
Configuration 3	5.0709	3.4484	65.97	0.0207
Configuration 3	14.9518	3.8474	66.56	0.0206
Configuration 3	42.12	3.9769	71.61	0.05716

**Table 4:** MXene/PVA nanocomposite results.

MXene/PVA	E Numerical (GPa)	E Experimental (GPa) [13]	EIM (GPa)	CLPT (GPa)
	[wt.% = 42.12]	[wt.% = 40]	[wt.% = 38.06]	[wt.% = 38.06]
Interface Layer EI (GPa)	Not considered		1 × 10^−3^	3.25 × 10^3^
Configuration 2	1.4414	3.7	43.777	5.8876
Configuration 3	42.12			

## Appendix A

Implementation of EIM: The implementation of EIM equations used in the paper are given here. Equation (A1) provides the overall nanocomposite stiffness matrix C. Here, the filler-matrix-filler interface and filler-matrix interface are assumed to have the same properties. The tensor T_fi_ is the dilute strain concentration tensor of the filler considering the interface in the matrix and T_f_ is the dilute strain concentration tensor of the filler alone and these are given in Equations (A2) and (A3). V_i_, V_f_ and V_m_ are the volume fractions of the interface, filler, and matrix, respectively. This interface volume fraction has been made a function of the filler volume fraction considering the interface to be layers above and below each MXene monolayer, as shown in Figure 2 and Figure 4 in the paper. The interface is modeled by using an equivalent continuum approach there by providing the equations given below:

(A1) $$C=C_{m}+\left[\left(V_{f}+V_{i}\right)\left(C_{i}\;-{\;C}_{m}\right)+V_{f}\left(C_{f}\;-{\;C}_{i}\right)T_{f}\right]\left[\left(V_{m}I+(V_{f}+V_{i}\right)T_{\mathrm{fi}}\right){]}^{-1}$$

(A2) $$T_{f}\;=I\;-{\;S}_{f}\;{\left[S_{f}+C_{m}{\left(C_{f}\;-{\;C}_{m}\right)}^{-1}\right]}^{-1}$$

(A3) $$T_{\mathrm{fi}}\;=I\;-{\;S}_{f}\left\{\left(\frac{V_{f}}{V_{i}+V_{f}}\right){\left[S_{f}+C_{m}{\left(C_{f}\;-{\;C}_{m}\right)}^{-1}\right]}^{-1}\;+\left(\frac{V_{i}}{V_{i}+V_{f}}\right){\left[S_{f}+C_{m}{\left(C_{i}\;-{\;C}_{m}\right)}^{-1}\right]}^{-1}\right\}$$

## Appendix B

Implementation of CLPT: The displacement and strain fields of the Kirchhoff’s plate theory are given by Equations (A4)–(A6) and Equations (A7)–(A10), respectively. Displacements at any point on the plate are u_1_(x_1_,x_2_,x_3_), u_2_(x_1_,x_2_,x_3_) and u_3_(x_1_,x_2_,x_3_), and displacements of the normal material line are ū _1_(x_1_,x_2_,x_3_), ū_2_(x_1_,x_2_,x_3_) and ū _3_(x_1_,x_2_,x_3_), where (x_1_,x_2,_x_3_) are the positions along the orthogonal unit basis vector (b_1_, b_2_, b_3_) forming the user-defined coordinate axis. The rotations of the cross-section of the plate (φ_1_, φ_2_) are assumed to be derivatives of the out-of-plane (direction b3) displacement of the mid-plane of the plate. Based on these assumptions, displacement, strain, and stress fields of the laminate can be computed as shown in [51,58]. The in-plane stiffness (A), bending stiffness (D), and coupled stiffness (B) matrices are calculated based on Equations (A11)–(A13). Because of the 2D plate structure assumption, the matrices are reduced to a 3 × 3 matrix. The overall stiffness matrix (Q) is represented by Equation (A14). The constitutive relationship based on in-plane forces and deformation as well as bending moments and curvatures of the laminated stack can be represented by Equation (A15). N and M are in-plane forces per unit length and bending moment per unit length, while εo and κ are the mid-plane strains and curvature of the laminate stack. Permutation matrix (S) of Equation (A16) is introduced solely to change signs and reorder the curvatures in Equation (A15). Equation (A17) is the reduced stiffness matrix of each layer (can be of filler, matrix, or interface). The term with the notation (.)_ij_ indicates the layer number considering up to n layers (k = 1,2, 3, …, n). The in-plane stiffness matrix (A) depends on the thickness of each layer and this helps in considering any shape of the inclusion as discussed in Section 3.2. The vectors and matrices are represented with bold letters as per the notation followed. The matrix S called the permutation matrix is introduced solely for the purpose of sign conventions.

(A4) $$u_{1}\left(x_{1},x_{2},x_{3}\right)=\bar{u_{1}}\left(x_{1},x_{2}\right)-x_{3}\varphi_{1};\;\varphi_{1}=\;\left(\frac{\partial \bar{u_{3}}}{\partial x_{1}}\right)$$

(A5) $$u_{2}\left(x_{1},x_{2},x_{3}\right)=\bar{u_{2}}\left(x_{1},x_{2}\right)-x_{3}\varphi_{2};\;\varphi_{2}=\left(\frac{\partial \bar{u_{3}}}{\partial x_{2}}\right)$$

(A6) $$u_{3}\left(x_{1},x_{2},x_{3}\right)=\bar{u_{3}}\left(x_{1},x_{2}\right)$$

(A7) $$\gamma_{13}=\gamma_{23}=0$$

(A8) $$\epsilon_{1}=\frac{\partial \bar{u_{1}}}{\partial x_{1}}-x_{3}\varphi_{1}{}^{2};\varphi_{1}{}^{2}=\;\left(\frac{\partial^{2}\bar{u_{3}}}{\partial x_{1}^{2}}\right)\;$$

(A9) $$\epsilon_{2}=\frac{\partial \bar{u_{2}}}{\partial x_{2}}-x_{3}\varphi_{2}{}^{2};\varphi_{2}{}^{2}=\;\left(\frac{\partial^{2}\bar{u_{3}}}{\partial x_{2}^{2}}\right)$$

(A10) $$\gamma_{13}=\frac{\partial \bar{u_{1}}}{\partial x_{2}}+\;\frac{\partial \bar{u_{2}}}{\partial x_{1}}-\;2x_{3}\left(\frac{\partial^{2}\bar{u_{3}}}{\partial x_{1}\partial x_{2}}\right)$$

(A11) $$A\;=\left[\begin{array}{ccc} A_{11} & A_{12} & A_{13}\\ A_{21} & A_{22} & A_{23}\\ A_{31} & A_{32} & A_{33} \end{array}\right]$$

(A12) $$B\;=\left[\begin{array}{ccc} B_{11} & B_{12} & B_{13}\\ B_{21} & B_{22} & B_{23}\\ B_{31} & B_{32} & B_{33} \end{array}\right]$$

(A13) $$D\;=\left[\begin{array}{ccc} D_{11} & D_{12} & D_{13}\\ A_{21} & A_{22} & D_{23}\\ D_{31} & D_{32} & D_{33} \end{array}\right]\;$$

(A14) $$Q=\left[\frac{\begin{array}{ccc} A_{11} & A_{12} & A_{13}\\ A_{21} & A_{22} & A_{23}\\ A_{31} & A_{32} & A_{33} \end{array}\begin{array}{ccc} B_{11} & B_{12} & B_{13}\\ B_{21} & B_{22} & B_{23}\\ B_{31} & B_{32} & B_{33} \end{array}}{\begin{array}{ccc} B_{11} & B_{12} & B_{13}\\ B_{21} & B_{22} & B_{23}\\ B_{31} & B_{32} & B_{33} \end{array}\begin{array}{ccc} D_{11} & D_{12} & D_{13}\\ A_{21} & A_{22} & D_{23}\\ D_{31} & D_{32} & D_{33} \end{array}}\right]$$

(A15) $$\left\{\begin{array}{c} N\\ SM \end{array}\right\}\;=\left[\begin{array}{cc} A & B\\ B & D \end{array}\right]\;\left\{\begin{array}{c} \varepsilon_{o}\\ S\kappa \end{array}\right\}$$

(A16) $$S=\left[\begin{array}{ccc} 0 & 1 & 0\\ -1 & 0 & 0\\ 0 & 0 & -1 \end{array}\right]$$

(A17) $$C\;=\left[\begin{array}{ccc} C_{11} & C_{12} & C_{16}\\ C_{21} & C_{22} & C_{26}\\ C_{61} & C_{62} & C_{66} \end{array}\right]\;$$

## Appendix C

Element 75 of MSC Marc: Equations (A18)–(A22) (h is the single plate thickness) provides the strain expressions equations used to model the thick-plate elements of MXenes in numerical analysis. The displacement field are same as Equations (A4)–(A6).

(A18) $$\epsilon_{1}=\frac{\partial \bar{u_{1}}}{\partial x_{1}}+x_{3}\hat{\varphi_{2}};\;\hat{\varphi_{2}}=\;\left(\frac{\partial \varphi_{2}}{\partial x_{1}}\right)\;$$

(A19) $$\epsilon_{2}=\frac{\partial \bar{u_{2}}}{\partial x_{2}}-\;x_{3}\hat{\varphi_{1}};\;\hat{\varphi_{1}}=\;\left(\frac{\partial \varphi_{1}}{\partial x_{2}}\right)$$

(A20) $$\epsilon_{12}=\frac{1}{2}\left[\left(\frac{\partial \bar{u_{1}}}{\partial x_{2}}+\frac{\partial \bar{u_{2}}}{\partial x_{1}}\right)+\;x_{3}\left(\frac{\partial \varphi_{2}}{\partial x_{2}}\;-\;\frac{\partial \varphi_{1}}{\partial x_{1}}\right)\right]$$

(A21) $$\epsilon_{23}=\left(\frac{\partial \bar{u_{3}}}{\partial x_{2}}\;-\;\varphi_{1}\right)\left(1-\frac{4}{h^{2}}x_{3}{}^{2}\right)$$

(A22) $$\epsilon_{23}=\left(\frac{\partial \bar{u_{3}}}{\partial x_{1}}\;-\;\varphi_{2}\right)\left(1-\frac{4}{h^{2}}x_{3}{}^{2}\right)$$

Element 7 of MSC Marc: The displacement assumption and mapping from x-y-z space or b1-b2-b3 space into a cube (Element 7) in the ξ, η, ζ space is given below. Equations (A23) and (A24) are the coordinate transformation equations used in MSC Marc and provided by the developer [46].

(A23) $$x=a_{0}+a_{1}\xi +a_{2}\eta +a_{3}\zeta +a_{4}\xi \eta +a_{5}\zeta \xi +a_{6}\xi \zeta +a_{7}\zeta \xi \eta$$

(A24) $$\psi =b_{0}+b_{1}\xi +b_{2}\eta +b_{3}\zeta +b_{4}\xi \eta +b_{5}\zeta \xi +b_{6}\xi \zeta +b_{7}\zeta \xi \eta$$

Function or coordinate expressed through nodal quantities using integration function are given below. Equation (A25) provides the elemental to global displacement transformation and Equations (A26)–(A33) are the shape functions used in the process of the transformation:

(A25) $$x=\overset{8}{\underset{i=1}{\sum }}x_{i}\rho_{i}$$

(A26) $$\rho_{1}=\frac{1}{8}\left(1-\xi \right)\left(1-\eta \right)\left(1-\zeta \right)$$

(A27) $$\rho_{2}=\frac{1}{8}\left(1+\xi \right)\left(1-\eta \right)\left(1-\zeta \right)$$

(A28) $$\rho_{3}=\frac{1}{8}\left(1+\xi \right)\left(1+\eta \right)\left(1-\zeta \right)$$

(A29) $$\rho_{4}=\frac{1}{8}\left(1-\xi \right)\left(1+\eta \right)\left(1-\zeta \right)$$

(A30) $$\rho_{5}=\frac{1}{8}\left(1-\xi \right)\left(1-\eta \right)\left(1+\zeta \right)$$

(A31) $$\rho_{6}=\frac{1}{8}\left(1+\xi \right)\left(1-\eta \right)\left(1+\zeta \right)$$

(A32) $$\rho_{7}=\frac{1}{8}\left(1+\xi \right)\left(1+\eta \right)\left(1+\zeta \right)$$

(A33) $$\rho_{8}=\frac{1}{8}\left(1-\xi \right)\left(1+\eta \right)\left(1+\zeta \right)$$
